# Higher Serum Levels of Lactate Dehydrogenase Before Microsurgery Predict Poor Outcome of Aneurysmal Subarachnoid Hemorrhage

**DOI:** 10.3389/fneur.2021.720574

**Published:** 2021-08-12

**Authors:** Shufa Zheng, Haojie Wang, Guorong Chen, Huangcheng Shangguan, Lianghong Yu, Zhangya Lin, Yuanxiang Lin, Peisen Yao, Dezhi Kang

**Affiliations:** ^1^Department of Neurosurgery, Neurosurgery Research Institute, The First Affiliated Hospital, Fujian Medical University, Fuzhou, China; ^2^Fujian Key Laboratory of Precision Medicine for Cancer, The First Affiliated Hospital, Fujian Medical University, Fuzhou, China; ^3^Key Laboratory of Radiation Biology of Fujian Higher Education Institutions, The First Affiliated Hospital, Fujian Medical University, Fuzhou, China

**Keywords:** serum lactate dehydrogenase level, aneurysmal subarachnoid hemorrhage, risk factor, biomaker, outcome

## Abstract

**Introduction:** We explored whether higher preoperative serum levels of lactate dehydrogenase (LDH) predicted outcome 3 months after surgery in patients with aneurysmal subarachnoid hemorrhage (aSAH) treated using microsurgical clipping in our institution.

**Methods:** Patients with aSAH treated at our institution between 2010 and 2018 were enrolled. The following parameters were recorded: age, sex, smoking and drinking history, medical history, Hunt–Hess and Fisher grades, aneurysm location, aneurysm size, surgical treatment, delayed cerebral ischemia (DCI), intracranial infection, hydrocephalus, pneumonia, and preoperative serum LDH levels within 24 h of aSAH. We investigated whether preoperative serum LDH levels were associated with Hunt–Hess grade, Fisher grade, and functional neurological outcome.

**Results:** In total, 2,054 patients with aSAH were enrolled, 874 of whom were treated using microsurgical clipping. The average serum LDH level (U/L) was significantly lower in the good outcome group (180.096 ± 50.237) than in the poor outcome group (227.554 ± 83.002; *p* < 0.001). After propensity score matching, the average serum LDH level (U/L) was still lower in the good outcome group (205.356 ± 76.785) than in the poor outcome group (227.119 ± 86.469; *p* = 0.029). The area under the receiver operating characteristic (ROC) curve was 0.702 (95% confidence interval [CI]: 0.650–0.754; *p* < 0.001). Based on the ROC curve, the optimal cutoff value for serum LDH levels as a predictor of poor 3-month outcome (modified Rankin Scale score > 2) was 201.5 U/L. The results revealed that Hunt–Hess grade, Fisher grade, DCI, pneumonia, and serum LDH (>201.5 U/L) were significantly associated with poor outcome. After propensity score matching, serum LDH levels > 201.5 U/L were still considered an independent risk factor for poor outcome (odds ratio: 2.426, 95% CI = 1.378–4.271, *p* = 0.002). Serum LDH levels were associated with Hunt–Hess and Fisher grades and were correlated with functional neurological outcomes (*p* < 0.001).

**Conclusions:** Our findings showed that higher preoperative serum levels of LDH correlated with Hunt–Hess grade, Fisher grade, and neurological functional outcome, and predicted the outcome of aSAH treated by microsurgical clipping at 3 months, which was involved in the related mechanisms of early brain injury and showed its potential clinical significance in patients with aSAH.

## Introduction

Lactate dehydrogenase (LDH) is a glycolytic enzyme that occurs in all important human organs, including the liver, heart, skeletal muscle, kidney, lung, and brain (1, 2). It catalyzes the dehydrogenation of lactic acid to pyruvic acid, promotes anaerobic glycolysis, and prevents lactic acid accumulation; the latter are associated with unfavorable clinical outcomes of traumatic brain injury (3). When cytolysis occurs or the cell membrane is destroyed, LDH is released into the blood, resulting in an increase in serum LDH (4). LDH activity can be detected in malignant tumor tissues and leukemic cells (5), and serum LDH levels are correlated with the prognosis of adult T-cell leukemia-lymphoma (6), prostate cancer (7), acute myeloid leukemia (8), melanoma (9), neuroblastoma (10), glioblastoma multiforme (11), acute encephalopathy (12), and *Mycoplasma pneumoniae* pneumonia (13). Serum LDH levels reflect the degree of brain tissue injury, and Yu et al. demonstrated that serum LDH activity is associated with middle cerebral artery occlusion in a dose-dependent manner (14). Several reports have shown that LDH quantification predicts neuronal injury (15, 16) and may predict poor prognosis of traumatic brain injury (17) and neonatal intracranial hemorrhage (18).

Several risk factors contribute to the poor prognosis in aneurysmal subarachnoid hemorrhage (aSAH), such as hypertension, poor Hunt–Hess grade, higher Fisher grade, hydrocephalus, pneumonia, and treatment modalities (19–21). However, few reports have explored the clinical significance of serum LDH levels in patients with aSAH, and the role of LDH in aSAH has not been fully established. At least two sources may contribute to higher serum LDH levels in patients with aSAH: (1) apoptotic/necrotic/damaged neurons or glial cells, (2) lytic red blood cells (RBCs) after release into the cerebrospinal fluid (CSF). Lu et al. reported that the number of apoptotic/necrotic/damaged cells was positively correlated with clinical condition in patients with aSAH, as well as with their Hunt–Hess grade (22). Similarly, the number of RBCs in the cerebra cisterna, sulcus, and/or ventricle was correlated with Fisher grade. Frontera found that early brain ischemia injury was associated with worse Hunt–Hess grade, which indicates poor acute neurological status and is correlated with worse functional outcomes after SAH (23). Claassen et al. showed that SAH completely filling the cistern or fissure, as well as intraventricular hemorrhage (IVH) on computed tomography (CT), were risk factors for delayed cerebral ischemia (DCI) (24), which is correlated with poor outcomes after SAH. However, few reports have explored the relationship between serum LDH levels and the extent of cerebral tissue injury in patients with aSAH. In the present study, we explored the clinical significance of serum LDH in patients with aSAH treated using microsurgical clipping in our institution. We hypothesized that higher preoperative serum levels of LDH, which may be correlated with Hunt–Hess grade and Fisher grade, predict 3-month outcome in patients with aSAH treated using microsurgical clipping.

## Materials and Methods

### Participants

Patients were enrolled in the study based on the following criteria: (1) diagnosis of SAH confirmed by CT; (2) presence of intracranial aneurysms confirmed using CT angiography (CTA) or digital subtraction angiography (DSA); (3) all aneurysms treated using microsurgical clipping; (4) CTA and/or DSA performed postoperatively. (5) patients were admitted 24 h after the onset of SAH. The exclusion criteria were as follows: (1) aSAH detected > 24 h after occurrence; (2) other cerebrovascular diseases (such as cerebral arteriovenous malformations, intracranial arteriovenous fistula, or moyamoya syndrome/disease) or intracranial tumors; (3) history of myocardial infarction, hepatitis, malignant tumor, pulmonary infarction, leukemia, hemolytic anemia, kidney disease, or progressive muscular atrophy. The data of patients with aSAH at our institution between 2010 and 2018 were collected. The following parameters were recorded: age, sex, smoking and drinking history, medical history (hypertension, diabetes, coronary heart disease, cerebral stroke), Hunt–Hess and Fisher grades, aneurysm location, aneurysm size, surgical treatment (conventional or decompressive craniotomy), delayed cerebral ischemia (DCI), intracranial infection, hydrocephalus, pneumonia, and preoperative serum LDH levels within 24 h of aSAH.

### Treatment

After confirmation, ruptured intracranial aneurysms were treated using microsurgical clipping within 72 h of aSAH onset. After surgery, patients were treated according to current aSAH guidelines (25), including prevention of cerebral arterial narrowing, improvement of cerebral blood flow, neurotrophic treatment, stress ulcer prevention, and nutritional support.

### Follow-Up Visit and Definition of Outcome

Postoperative complications were evaluated using CT scanning within 24 h of surgery. The neurological outcome was assessed at the 3-month follow-up and classified according to the modified Rankin Scale (mRS) score. Good clinical outcome was defined as an mRS score of 0–2, while poor outcome was assigned to patients with an mRS score of 3–6. Functional outcome was divided into four levels according to mRS: no symptoms (mRS = 0), no significant to slight disability (mRS = 1–2), moderate to serious disability (mRS = 3–4), and severe disability to death (mRS = 5–6). To define the relationship between serum LDH levels and clinical outcome in patients with aSAH, we investigated whether preoperative serum LDH levels were associated with Hunt–Hess grade, Fisher grade, or the upper four functional outcomes.

### Statistical Analysis

All statistical analyses were performed using SPSS for Windows (version 25.0; IBM Corp., Armonk, NY, USA). The Kolmogorov–Smirnov test was used to check whether the data had a normal distribution. One-way analysis of variance or the Student's *t*-test were used to determine the significance of differences in continuous data. The χ^2^ test or Fisher's exact test was used to determine the significance of differences in qualitative data. Multivariable analysis was carried out using a backward stepwise logistic regression model that included all variables with a *p*-value of <0.10 in the univariate analysis. In the multivariable analysis model, age was divided into “ ≤ 60 years” and “> 60 years” (26), Hunt–Hess grade into “low grade” (I–III) and “high grade” (IV–V), Fisher grade into “low grade” (1–3) and “high grade” (4), and serum LDH levels into “ ≤ optimal cutoff value” and “> optimal cutoff value.” Statistical significance was set at a *p*-value of <0.05. The receiver operating characteristics (ROC) curve (MedCalc for Windows version 15.2.2; Mariakerke, Belgium) was generated to analyze the specificity and sensitivity of serum LDH levels for mRS. Propensity-score matching (PSM) was performed to remove imbalances in basic clinical characteristics between the good outcome and poor outcome groups, as well as between the pneumonia and non-pneumonia groups. Conditional probability was estimated using a logistic regression model. The good outcome and poor outcome groups were matched at a ratio of 1:1 using the nearest neighboring matching algorithm.

## Results

**Figure 1:**
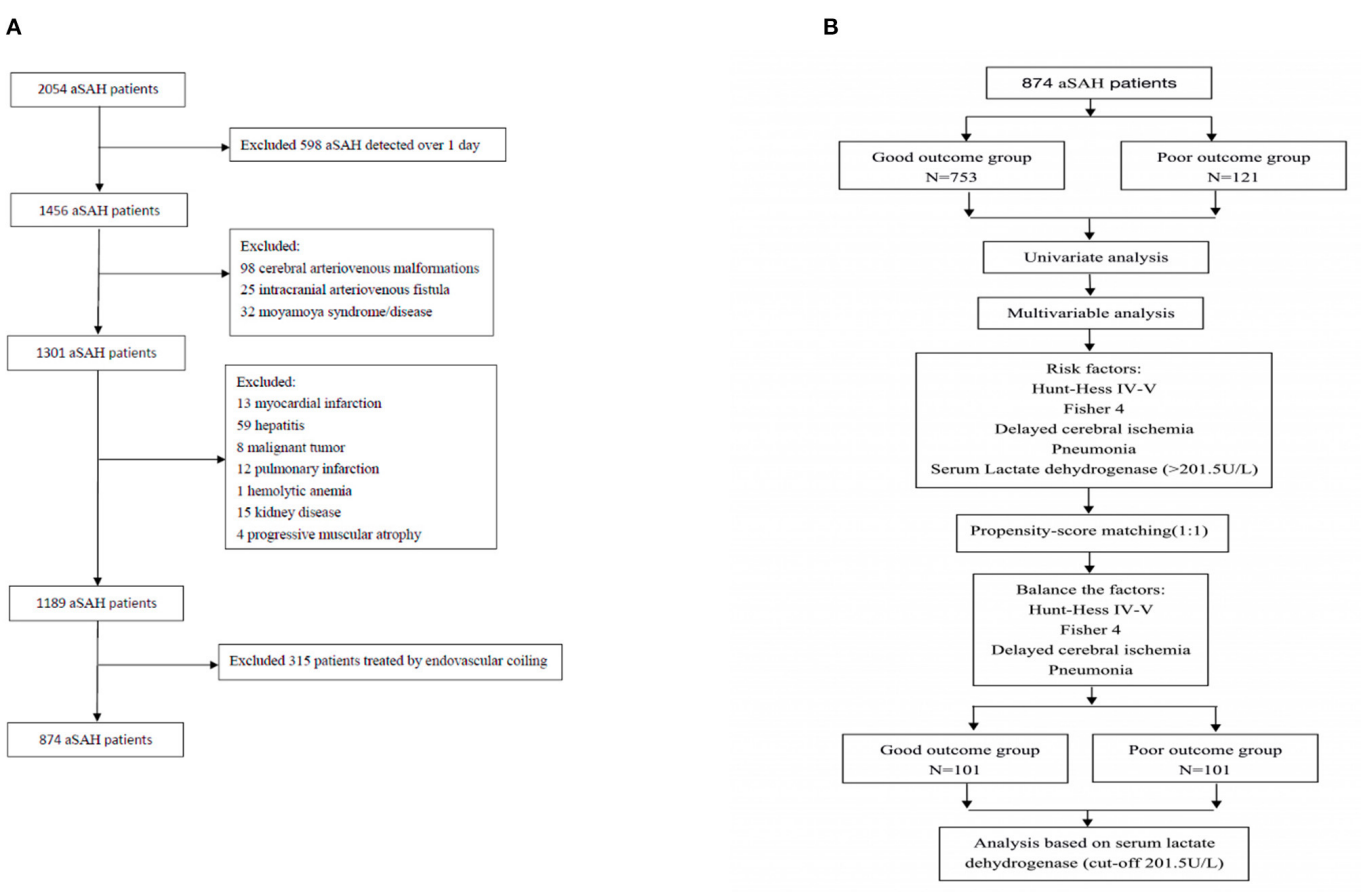
**(A)** Flowchart of patient inclusion; **(B)** Flowchart of propensity-score matching.

**Figure 2:**
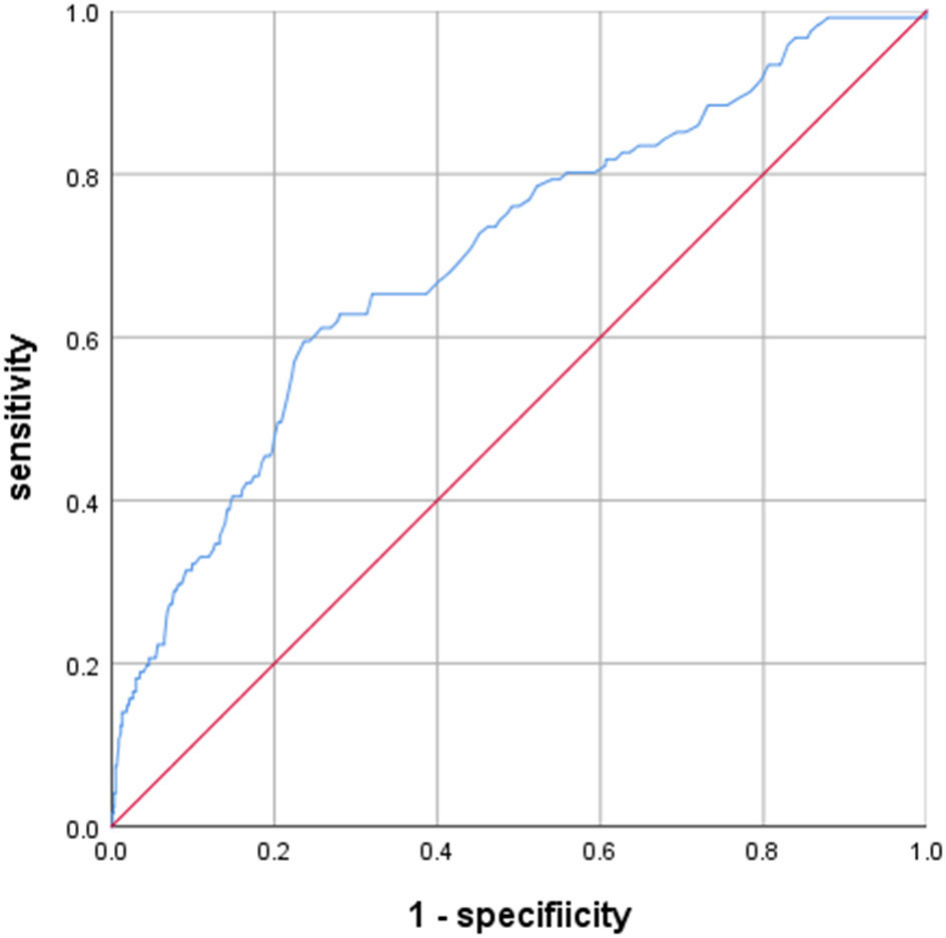
Predictive values of LDH for 3-month modified Rankin Scale (mRS) >2 area under curve 0.702 (95% confidence interval [CI], 0.650–0.754; *p* < 0.001).

A total of 2,054 patients with aSAH were treated in our institution between 2010 and 2018, and 874 patients treated using microsurgical clipping were enrolled based on the above criteria (Figure 1). The incidence of poor outcomes following aSAH was 13.8% (121/874). The basic clinical characteristics of patients with aSAH are shown in Tables 1, 2. The average serum LDH level (U/L) was significantly lower in the good outcome group (180.096 ± 50.237) than in the poor outcome group (227.554 ± 83.002; *p* < 0.001). After PSM, the average serum LDH level (U/L) was still lower in the good outcome group (205.356 ± 76.785) than in the poor outcome group (227.119 ± 86.469; *p* = 0.029). The ROC curve of serum LDH levels for poor outcome in patients with aSAH at the 3-month follow-up is shown in Figure 2. The area under the ROC curve (AUC) was 0.702 (95% confidence interval [CI]: 0.650–0.754; *p* < 0.001). The optimal cutoff value for serum LDH levels as a predictor of poor 3-month outcome (mRS > 2) was determined to be 201.5 U/L based on the ROC curve. At this level, the sensitivity was 59.5% and the specificity 76.4%.

**Table 1:** Basic clinical characteristics of patients with aneurysmsal subarachnoid hemorrhage before and after propensity-score matching.

	**Before propensity-score matching**			**After propensity-score matching**		
**General information**	**Good outcome**	**Poor outcome**	***P*-value**	**Good outcome**	**Poor outcome**	***P*-value**
	**(*n* = 753)**	**(*n* = 121)**		**(*n* = 101)**	**(*n* = 101)**	
Age			0.043			0.128
≤ 65 yrs	645	95		74	83	
>65 yrs	108	26		27	18	
Age range (y)	10–86	22–85		10–85	22–85	
Sex			0.193			0.240
Male	302	41		40	32	
Female	451	80		61	69	
Smoking	115	7	0.593	11	6	0.205
Drink	75	7	0.144	9	5	0.268
Medical history						
Hypertension	327	73	0.001	61	59	0.774
Diabetes	40	11	0.100	8	7	0.788
Coronary heart disease	9	2	0.675	2	1	0.561
Cerebral stroke	13	2	0.954	2	1	0.561
Hunt-Hess grade			0.000			0.196
0-III	677	58		65	56	
IV-V	76	63		36	45	
Fisher			0.000			0.396
1–3	621	57		59	53	
4	132	64		42	48	
Location of Aneurysm						
Internal carotid artery	141	25	0.614	12	20	0.123
Anterior choroidal artery	25	4	0.994	2	3	0.651
Ophthalmic artery	18	2	0.615	0	2	0.155
Posterior communicating artery	160	24	0.723	25	19	0.306
Middle cerebral artery	171	33	0.271	23	29	0.334
Anterior communicating artery	230	47	0.069	37	41	0.563
Basilar artery	4	3	0.026	0	1	0.316
Anterior cerebral artery	49	6	0.515	8	3	0.121
Posterior cerebral artery	6	2	0.359	0	2	0.155
Aneurysm size			0.510			0.731
<5 mm	451	83		69	76	
5–15 mm	328	55		32	37	
15–25 mm	19	6		5	7	
>25 mm	6	2		1	0	
Surgical treatment			0.246			0.346
Conventional craniotomy	711	111		89	93	
Decompressive craniotomy	42	10		12	8	
Delay ischemic neurological deficit	74	43	0.000	29	30	0.877
Hydrocephalus	117	54	0.000	38	40	0.773
Intracranial infection	53	13	0.152	12	11	0.825
Pneumonia	148	79	0.000	65	61	0.561
Serum Lactate dehydrogenase (>201.5U/L)	178	72	0.000	38	60	0.002

**Table 2:** Predictors for poor outcome of aSAH in multivariable model.

	**Univariate analysis**				**Multivariable analysis**				**After propensity-score matching**			
	**OR (95%CI)**				**AOR (95%CI)**				**OR (95%CI)**			
**Independent Variable**	**OR**	**lower**	**upper**	***P*-value**	**AOR**	**lower**	**upper**	***P*-value**	**OR**	**lower**	**upper**	***P*-value**
Age	1.025	1.008	1.043	0.004	1.019	0.998	1.040	0.075				
Hypertension	1.981	1.339	2.931	0.001	0.876	0.528	1.451	0.606				
Diabetes	1.782	0.888	3.578	0.104	1.056	0.436	2.557	0.904				
Hunt-Hess IV-V	2.746	2.244	3.360	0.000	1.637	1.266	2.118	0.000				
Fisher 4	2.445	1.994	2.998	0.000	1.517	1.182	1.946	0.001				
Anterior communicating artery aneurysm	1.444	0.971	2.148	0.070	1.048	0.636	1.727	0.855				
Basilar artery aneurysm	3.803	0.897	16.125	0.070	3.296	0.479	22.693	0.226				
Delayed cerebral ischemia	5.058	3.248	7.877	0.000	4.234	2.412	7.432	0.000				
Hydrocephalus	4.381	2.910	6.596	0.000	1.043	0.612	1.778	0.877				
Pneumonia	7.689	5.076	11.646	0.000	3.848	2.386	6.206	0.000				
Serum Lactate dehydrogenase (>201.5U/L)	4.747	3.182	7.081	0.000	2.702	1.645	4.440	0.000	2.426	1.378	4.271	0.002

To analyze the predictors of poor outcome in patients with aSAH, the following variables with a significance level of *p* < 0.10 were included in a univariate analysis: age, hypertension, diabetes, Hunt–Hess grade, Fisher grade, anterior communicating artery aneurysm, basilar artery aneurysm, DCI, hydrocephalus, pneumonia, and serum LDH levels of > 201.5 U/L. The results revealed that age, hypertension, Hunt–Hess grade, Fisher grade, DCI, hydrocephalus, pneumonia, and serum LDH levels > 201.5 U/L were associated with poor 3-month outcomes (Table 2; *p* < 0.05). In a multivariable analysis, Hunt–Hess grade, Fisher grade, DCI, pneumonia, and serum LDH levels >201.5 U/L were still significantly associated with outcome, whereas age, hypertension, diabetes, anterior communicating artery aneurysm, basilar artery aneurysm, and hydrocephalus were not. Patients with a Hunt–Hess grade of IV–V had a 1.6-fold increased risk of poor outcomes (odds ratio [OR]: 1.637; 95% CI: 1.266–2.118, *p* < 0.001). Those with a Fisher grade of 4 had a 1.5-fold increased risk of poor outcomes (OR: 1.517, 95% CI: 1.182–1.946, *p* = 0.001). DCI conferred a 4.2-fold increased risk of poor outcomes (OR: 4.234, 95% CI: 2.412–7.432, *p* < 0.001). Pneumonia was associated with a 3.8-fold increased risk of poor outcomes (OR: 3.848, 95% CI: 2.386–6.206, *p* < 0.001). Serum LDH levels >201.5 U/L showed a 2.7-fold increased risk of poor outcomes (OR: 2.702, 95% CI: 1.645–4.440, *p* < 0.001; Table 2). After PSM, there were no significant differences in Hunt–Hess grade, Fisher grade, DCI, or pneumonia between the good outcome and poor outcome groups (Tables 1, 2). In the logistic regression model (Table 2), serum LDH levels >201.5 U/L were still considered an independent risk factor for poor outcome (OR: 2.426, 95% CI: 1.378–4.271, *p* = 0.002).

**Figure 3:**
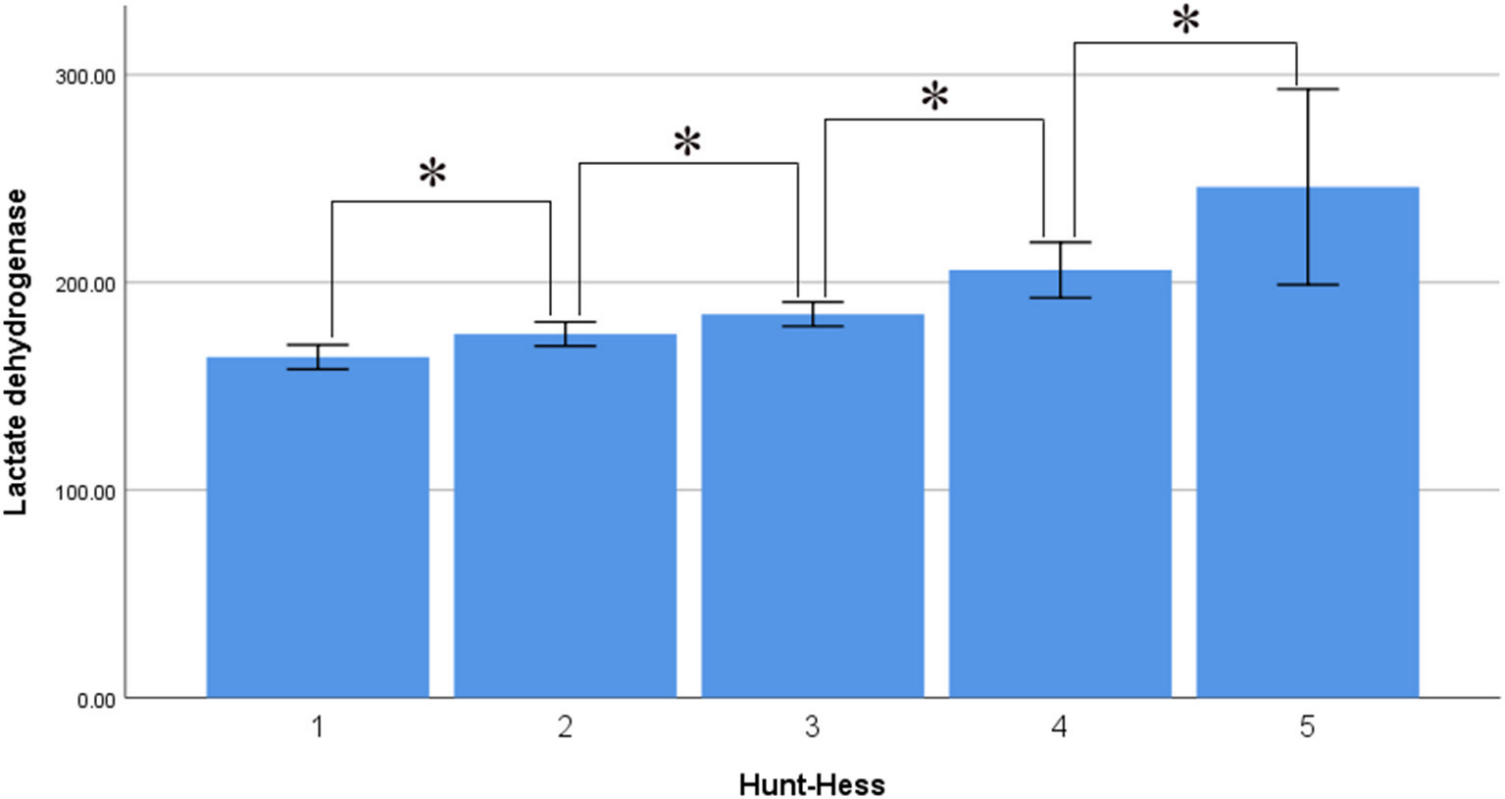
Average level of serum LDH in different Hunt-Hess grade (asterisk represent statistically significant differences).

**Figure 4:**
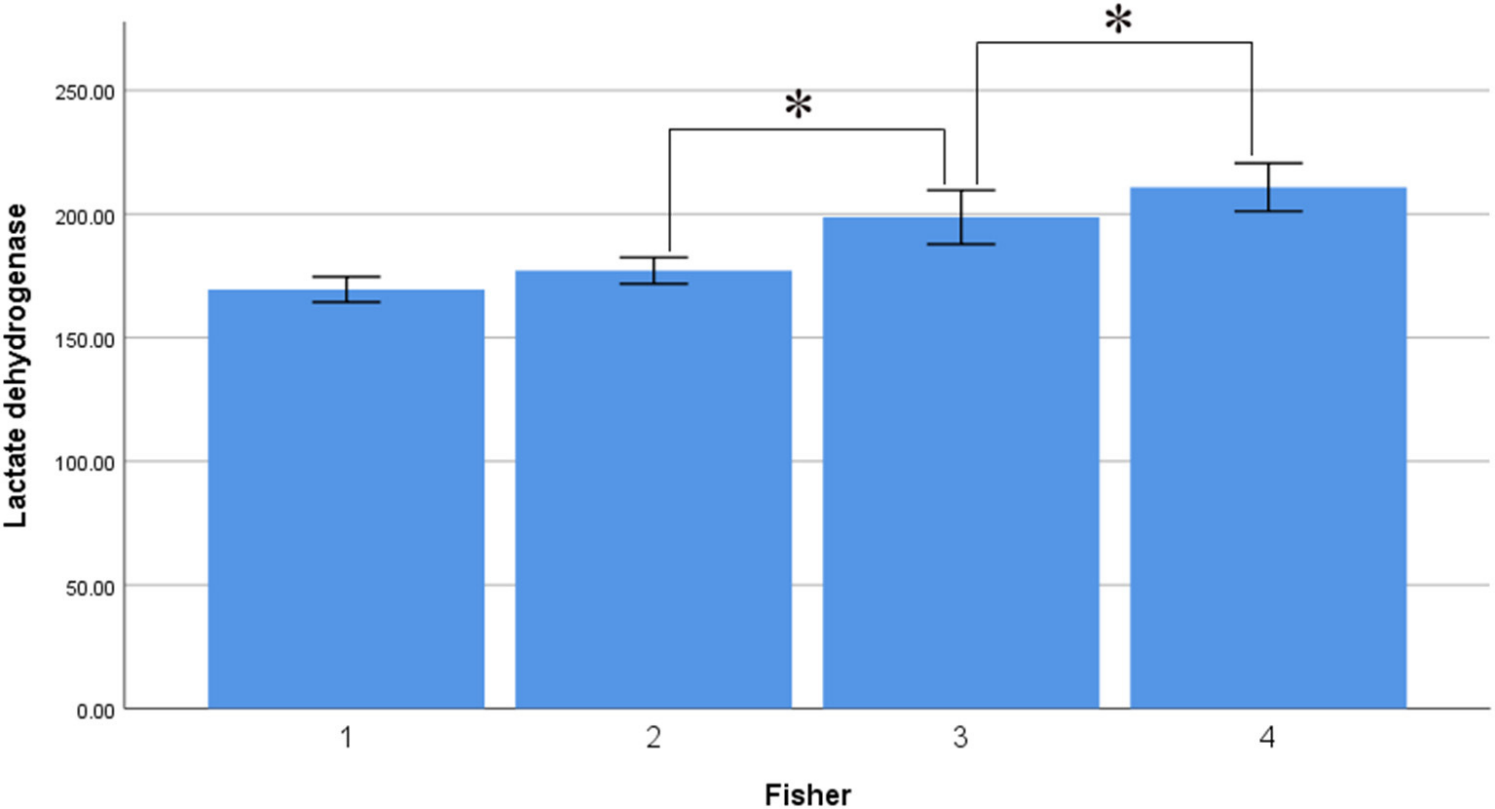
Average level of serum LDH in different fisher grade (asterisk represent statistically significant differences).

Interestingly, serum LDH levels were associated with Hunt–Hess and Fisher grade, with levels of 163.880 ± 35.571 U/L in the Hunt–Hess grade I group, lower than those in the grade II (174.981 ± 49.616), III (188.306 ± 50.702), IV (225.609 ± 69.509), and V groups (252.851 ± 93.302). There were significant differences among the groups in this regard (*p* < 0.001), and there was a marked trend whereby serum LDH levels increased alongside Hunt–Hess grade (Figure 3). Serum LDH levels were 169.492 ± 41.621 in the Fisher grade 1 group, lower than in the grade 2 (177.097 ± 42.621), grade 3 (198.709 ± 72.553), and grade 4 groups (210.811 ± 68.962). There were statistically significant differences between grades 4 and 3, grades 4 and 2, grades 4 and 1, grades 3 and 2, grades 3 and 1 (*p* < 0.001 in all cases). There was a marked trend whereby serum LDH levels increased alongside Fisher grade (Figure 4).

**Figure 5:**
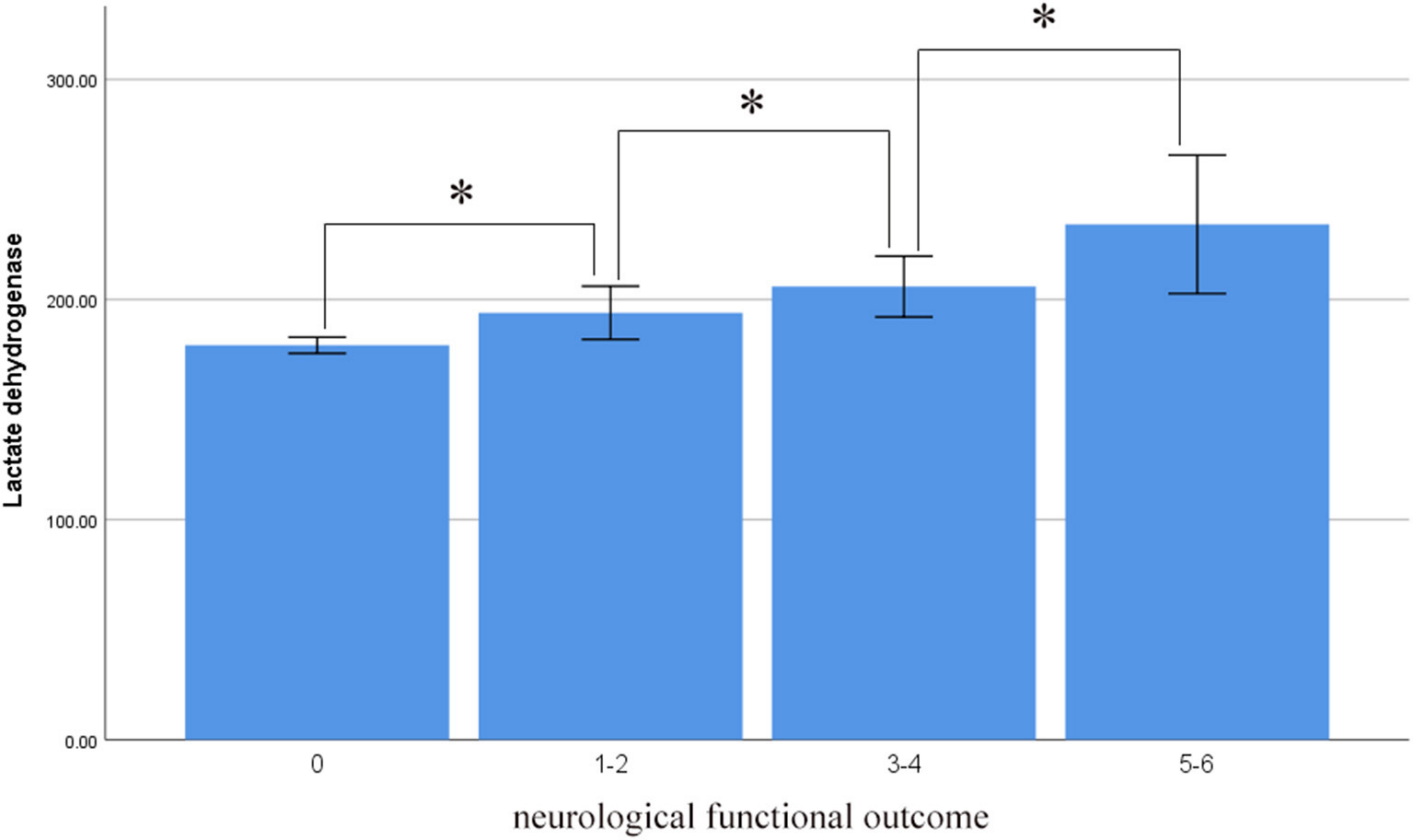
Average level of serum LDH in different neurological functional outcome (asterisk represent statistically significant differences).

Serum LDH levels were also correlated with functional neurological outcome at the 3-month follow-up. The serum LDH levels were 179.247 ± 46.761 in the mRS 0 group, lower than in the no significant to slight disability (mRS 1–2; 193.977 ± 69.399), moderate to serious disability (mRS 3–4; 205.918 ± 59.203), and severe disability to death groups (mRS 5–6; 234.188 ± 108.336). There were significant differences among the groups in this regard (*p* < 0.001). There was a marked trend whereby serum LDH levels increased as neurological function deteriorated (Figure 5).

## Discussion

Our findings showed a marked trend whereby serum LDH levels increased alongside Hunt–Hess and Fisher grades. In addition, Hunt–Hess grade, Fisher grade, DCI, pneumonia, and higher serum LDH levels predicted and contributed to poor outcome in patients with aSAH at 3 months. The optimal cutoff value for serum LDH levels as a predictor of 3-month poor outcome (mRS > 2) was 201.5 U/L. Moreover, serum LDH levels were correlated with functional neurological outcomes. There was a marked trend whereby serum LDH levels increased as neurological function deteriorated. After PSM, serum LDH (>201.5 U/L) was still considered an independent risk factor of poor outcome.

Serum LDH levels are correlated with the prognosis of adult T-cell leukemia-lymphoma (6), prostate cancer (7), acute myeloid leukemia (8), melanoma (9), neuroblastoma (10), glioblastoma multiforme (11), acute encephalopathy (12), and *Mycoplasma pneumoniae* pneumonia (13). However, few studies have investigated the relationship between LDH and aSAH. Regional cerebral blood flow and arteriovenous difference of oxygen are reduced due to primary aSAH injury (27), and cerebral ischemia causes an anaerobic shift of metabolism, leading to lactic acidosis and upregulation of serum LDH levels (28). There is a significant correlation between serum LDH and lactic acid levels, and both reflect the degree of tissue damage (27, 29).

Serum LDH levels can also reflect the severity of brain tissue injury. Neuronal apoptosis and necrosis have been observed 24 h after SAH (30, 31) and can result in cytolysis and cell membrane destruction. Subsequently, LDH is released into the blood from the damaged or dead cells, resulting in increased serum LDH (4). In the study by Yu et al. (14), serum LDH activity was associated with infarct volume and degree of middle cerebral artery occlusion in a dose-dependent manner. Several studies have shown that LDH can be quantified to predict neuronal damage (15, 16), and inhibition of LDH release may reduce neuronal apoptosis (14). Rao et al. reported that a significant increase in serum LDH levels was a predictor of severe brain damage and poor prognosis of traumatic brain injury (17), while Engelke et al. indicated that LDH was significantly correlated with subsequent seizures, hydrocephalus, and adverse long-term outcomes of neonatal intracranial hemorrhage (18).

In the present study, serum LDH levels increased alongside Hunt–Hess grade. We deduced that serum LDH levels were correlated with Hunt–Hess grade, and that they reflected the degree of early brain injury and the clinical condition of patients with aSAH. Furthermore, there was a marked trend whereby serum LDH levels increased as neurological function at the 3-month follow-up deteriorated. Subarachnoid clots in sulci/fissures induce spreading depolarizations and acute cerebral infarction of the adjacent cortex after cerebral aneurysm rupture (30). This is a mechanism of early brain injury after SAH (32) and contributes to the clinical condition of patients with aSAH. In a study by Frontera et al. (23), early ischemic brain injury was related to worse Hunt–Hess grade, higher rates of death, and severe disability/death (mRS 4–6) at the 3-month follow-up. Increased ischemic lesion volume has been associated higher Hunt–Hess grade and 3-month mRS (33), corroborating our own findings.

Our findings also showed a marked trend whereby serum LDH levels increased alongside Fisher grade. Fisher grade is higher when there is more blood in the subarachnoid space, and higher Fisher grade correlates with poor aSAH outcome (34, 35). After cerebral aneurysm rupture, the blood brain barrier is destroyed and RBCs are released into the subarachnoid space from the artery. These RBCs break down in the cerebrospinal fluid (CSF) (35) and LDH from the lysed RBCs is absorbed into the blood after being released into the CSF (17), increasing serum LDH levels. Therefore, higher serum LDH levels are associated with higher Fisher grade, which is closely related to poor outcome in patients with aSAH (34, 36, 37).

However, our study had some limitations. Firstly, LDH occurs in all important human organs and lacks specificity to the central nervous system. LDH was not collected and measured in the CSF in our patients, and serum LDH levels do not directly reflect the true levels in brain tissue. Secondly, imaging data were not available to confirm the relationship between serum LDH levels and the degree of brain tissue damage. Thirdly, serum LDH levels were within the normal range in some patients with poor outcomes, so they do not fully explain these patients' prognosis; the detailed mechanism needs further exploration.Fourthly, LDH, like C-reactive protein, neutrophil to lymphocyte ratio and white blood cell count, are related to the prognosis of SAH. They are biomarkers of the prognosis of SAH. However, the specific mechanism of LDH is still unclear. And there is still no effective treatment to reduce the mortality and disability rate of aSAH patients.

## Conclusions

Our findings showed that higher preoperative serum levels of LDH correlated with Hunt–Hess grade, Fisher grade, and functional neurological outcome, and that they predicted the 3-month outcome in patients with aSAH, which is associated with mechanisms of early brain injury and may have clinical significance in patients with aSAH.

## Data Availability Statement

The raw data supporting the conclusions of this article will be made available by the authors, without undue reservation.

## Ethics Statement

The studies involving human participants were reviewed and approved by Ethics Committee of the First Affiliated Hospital of Fujian Medical University. Written informed consent from the participants' legal guardian/next of kin was not required to participate in this study in accordance with the national legislation and the institutional requirements.

## Author Contributions

SZ, HW, GC, HS, and LY: acquisition of data and critical revision of manuscript for intellectual content. YL: study supervision. ZL, PY, and DK: study concept and design. PY and DK: analysis and interpretation of data and study supervision. All authors reviewed the manuscript.

## Conflict of Interest

The authors declare that the research was conducted in the absence of any commercial or financial relationships that could be construed as a potential conflict of interest.

## Publisher's Note

All claims expressed in this article are solely those of the authors and do not necessarily represent those of their affiliated organizations, or those of the publisher, the editors and the reviewers. Any product that may be evaluated in this article, or claim that may be made by its manufacturer, is not guaranteed or endorsed by the publisher.
